# The Effect of Using Bovine Colostrum and Probiotics on Performance, Egg Traits, Blood Biochemical and Antioxidant Status of Laying Japanese Quails

**DOI:** 10.3390/ani13132166

**Published:** 2023-06-30

**Authors:** Reza Mokhtarian Asl, Ali Nobakht, Valiollah Palangi, Aristide Maggiolino, Gerardo Centoducati

**Affiliations:** 1 Department of Animal Science, Maragheh Branch, Islamic Azad University, Maragheh 55, Iran; mokhtarian.reza@yahoo.com; 2 Department of Animal Science, Faculty of Agriculture, Ege University, Izmir 35100, Türkiye; valiollah.palangi@ege.edu.tr; 3 Department of Veterinary Medicine, University of Bari A. Moro, 70010 Valenzano, Italy

**Keywords:** antioxidant status, blood parameters, carcass characteristics, egg traits, Japanese quails

## Abstract

**Simple Summary:**

In addition to proteins and carbohydrates, colostrum contains antibodies. Quails can benefit from the consumption of colostrum as a source of probiotics and as a reduction in antibiotic usage, which helps improve their performance and reduce their environmental impact. In the current study, we found that the continuous use of bovine colostrum (BC) in the diet of laying Japanese quails during the late laying period improved egg production performance, egg traits, carcass characteristics, blood biochemistry, and antioxidant status.

**Abstract:**

The present paper aims to evaluate the effect of different levels of bovine colostrum and probiotic dietary supplementation on egg production performance, egg traits, carcass characteristics, blood biochemistry and antioxidant status of laying Japanese quails. For the trial, 240 laying quails, aged between 24 weeks and 30 weeks, were involved in a 3 × 2 factorial experimental design, with 3 levels of bovine fresh colostrum (0, 2, and 4 percent of the total ratio) and 2 levels of probiotics (0 and 0.01 percent of the total ratio) administration. The colostrum supplementation improved the egg production performance, egg traits, carcass characteristics, blood biochemistry, and antioxidant status (*p* < 0.01). Probiotics used without colostrum did not affect the investigated traits of laying Japanese quails (*p* > 0.05), but a synergistic effect was observed when combined with colostrum. The overall results recommended that using 4% of bovine colostrum in laying Japanese quails, with the addition of 0.01% of probiotic feed additive results in positive effects on egg production performance, egg traits, carcass characteristics, blood biochemistry, and antioxidant status of laying Japanese quails in the late laying period.

## 1. Introduction

The poultry industry faces a high market demand for food production. The most common animal source of food, consumed at a global level, is represented by poultry eggs and meat, probably because they meet the needs of a wide diversity of cultures and traditions [1]. Moreover, within the livestock sector, it is the most efficient livestock in terms of natural resources utilization, providing animal protein to supply the growing global demand.

Thus, even though a lot of research is being done in order to increase the productivity and efficiency of poultry throughout the world, it is essential that this increase in production and efficiency does not compromise the quality of meat and eggs, as well as the welfare of the poultry [2]. Researchers have tried many processes for improving animal performances and enhancing the quality of animal-origin foods, particularly via feed supplementation with highly attractive phytochemicals (for example, the use of oil compounds of wild plants and extracts of herbs, thyme and rosemary used for turkeys) bioactive phytochemicals are polyphenols, alkaloids, carotenoids, and flavonoids, along with tannins, terpenes, and saponins, which play vital roles in reducing disease-causing agents. Phytochemicals exhibit antioxidant, antimicrobial, antifungal, and anti-inflammatory properties as well as anti-parasitic and antiprotozoal properties [3,4]. Phytochemicals found in plants and mushrooms have been extensively studied for their antioxidant and anti-inflammatory properties. In recent years, phytochemicals have gained attention because of their ability to inhibit inflammation and induce antioxidant action. Excessive production of free radicals and oxidative stress are very common problems in commercial chicken production. As a result, natural antioxidants should be included as an essential factor in poultry nutrition [5,6]. This has led to their wide application in a variety of pathological conditions in human research, supporting studies on farm animals that aim to uncover their crosstalk.

The Japanese quail (*Coturnix japonica*) is diffused and bred for different purposes around the world, such as fighting, singing, or as a decorative bird as well as for eggs and meat due to its high productivity, feed conversion ratio (FCR), and easy breading [7,8]. In the past, antibiotics were often used to prevent disease and promote growth, but these practices have been banned both in Europe and the US. [9]. Many studies have proposed natural additives (cinnamon (such as: *Cinnamomum zeylanicum*) oil, mannan-oligosaccharides, humate, oregano, Yukashidigra extract, thyme vulgaris, ginger essence, exogenous enzymes, and propionic acid) [10,11] with antioxidant and immunomodulatory effects in poultry as probiotics, prebiotics, enzymes, herbs, and essential oils [12,13].

Bovine colostrum is the first secretion of mammals’ mammary glands after parturition, and it is known as a nutrient-dense fluid [14] containing several immune-regulating components, transferrin, essential and non-essential amino acids, insulin-like growth factor-I (IGF-1) and II (IGF-II), fatty acids, anti-microbials, immunoglobulins (Igs), enzymatic (lactoperoxidase, catalase, superoxide dismutase and glutathione peroxidase) and non-enzymatic antioxidants (vitamin A, C, E, lactoferrin and selenium), growth factors, oligosaccharides, and glycoconjugates [15,16,17]. Colostrum plays a vital role in feeding neonatal infants, improving their survival and growth rates, boosting the immune system, and preventing some diseases (e.g., enteritis, respiratory disease, septicemia, joint infections, three-fold increase in diarrhea, salmonella, E-coli) [18,19,20]. Immunoglobulins in colostrum in the early hours after birth can be easily transferred through the intestinal intercellular pores, reach the bloodstream and enhance the immune response [21]. The high level of proteins essential amino acids (threonine, cystine, valine), non-essential (glutamic acid and proline), vitamins (A, D_3_, E, K_3_ and C) and minerals (potassium, sodium, iron, and copper) in colostrum, has made it a valuable food for growth and for improving health in calves [22,23].

Since the amount of colostrum produced by dairy cows is more than their calves’ needs, it could be used as feed additive [24]. Many authors reported the positive effects of its dietary inclusion in poultry, observing improved final body weight, carcass yield, feed efficiency, oxidative status, and positive changes in their intestinal cells’ morphology [25,26,27]. In addition, probiotic microflora-based food products are essential components of the diet. Probiotics are live microorganisms that, when administered in adequate amounts, may confer a health benefit on the host. They can regulate intestinal microbial homeostasis, stabilize the gastrointestinal barrier function and the expression of bacteriocins (by increasing lactic acid and decreasing pH in the digestive system) [28,29], and modulate the defense system by improving the microbial flora [30] and interfering with other pathogens such as acute viral gastroenteritis and antibiotic-associated diarrhea (including Clostridium difficile infection) and Helicobacter pylori infection for mucosa colonization [31,32]. Probiotics are beneficial microbial products that are often added to poultry diets to improve their health, increase their performance, and improve carcass traits. Similar to other poultry species, in recent years, the use of beneficial microbial products (Protexin^®^, Fermacto^®^) as probiotics in quails has been developed [33]. The use of probiotics in young quail diets has had different results on the modification of the intestinal microbiota, stimulation of the immune system, reduction of inflammatory reactions, prevention of pathogen colonization, enhancement of growth performance, increase of intestinal digestibility and total tract apparent digestibility coefficient, and decrease in ammonia and urea excretion [34,35,36]. It has been shown in a report that the Protoxin probiotic produced by the British company Probiotics International Ltd. can act as a potential alternative to antibiotic growth promoters in poultry production (maintaining the intestinal microflora and tissue regeneration acceleration) [37].

In previous studies, the positive effects of continuous and intermittent use of cow’s colostrum and determining the consumption of the minimum level of cow’s colostrum in the feeding of laying Japanese quails have been obtained [38], but so far, no research has been conducted on the evaluation of the effects of cow’s colostrum (a natural additive) and probiotics (an artificial additive) in laying Japanese quails. Therefore, the aim of this research is to evaluate the effects of different levels of cow’s colostrum, with and without probiotics, on performance, egg quality, carcass traits, blood parameters, and antioxidant status in Japanese quails.

## 2. Materials and Methods

### 2.1. Animals, Treatment, and Management

The Animal Welfare Committee of Islamic Azad University (Maragheh Branch) approved the animal care protocol used in this experiment (Protocol no. 1396-IAU. 21 May 2017).

In the current study, 240 laying quails were involved. All animals were randomly divided into 6 groups as 3 × 2 factorial arrangements containing 3 levels of bovine fresh colostrum, 0% (Col-0), 2% (Col-2) and 4% (Col4), and 2 levels of probiotic addition, 0% (Prob-0) and 0.01% (Prob-0.01). Treatments included: (1) colostrum 0% and probiotics 0%; (2) colostrum 0% and probiotics 0.01%; (3) colostrum 2% and probiotics 0%; (4) colostrum 4% and probiotics 0%; (5) colostrum 2% and probiotics 0.01%; (6) colostrum 4% and probiotics 0.01%. The percentage of inclusion in the total weight compared to the breeding standard of laying Japanese quails in Iran (approximately 180 cm^2^ area for each quail) was estimated. Each of the 6 experimental groups had 4 cages with dimensions of 45 × 50 cm and height of 25 cm. Each cage contained 10 laying Japanese quails (40 quails in each group). Each cage compartment was equipped with a nipple water bottle and a trough-type feeder. The room temperature was 22 °C, the birds were exposed to a 16 h/8 h light/dark illumination cycle, and the trial lasted 6 weeks, from 24 to 30 weeks of age. The colostrum was milked from three pluriparous Holstein cows at 6 h from calving, totally mixed and stored at −20 °C in bottles of 200 mL. The colostrum was analyzed in the food laboratory of the university where the research was conducted with a *LactoScan COMBO* device (made in Stara Zagora, Bulgaria) and was characterized by 27.5 g/100 g of DM, 6.7 g/100 g of fat, 16.9 g/100 g of protein, 2.6 g/100 g of lactose, and 1.3 g/100 g of ash. Each day, the colostrum was thawed in water at 30 °C and completely melted and mixed thoroughly with the whole diet. The prepared diets were stored in opaque polyethylene bags and kept in a cool (10–20 °C) and dry (35–40% humidity) environment until they were fed to experimental quails. Water at 2% inclusion was added in the control diet to simulate similar humidity inclusion in the diets. Diets were formulated using NRC quail nutrition tables [39] on the base of corn and soybean meal using UFFDA’s diet software (designed at the University of Georgia, Athens, GA, USA) (Table 1).

The probiotic used was a commercial one, Bio-Poul^®^ (Biodep, Unit 10, Corner of 39th Square, Farjam St., Resalat Sq., Tehran, Iran); it is a combination of 9 bacterial and yeast strains selected based on in vitro and in vivo tests and specially formulated for poultry (*Enterococcus faecium*, *Pediococcus acidilactici*, *Bacillus subtilis*, *Lactobacillus acidophilus*, *Lactobacillus plantarum*, *Lactobacillus rhamnosus*, *Lactobacillus casei*, *Bifidobacterium bifidum*, *Saccharomyces cerevisiae*), as well as calcium carbonate as filler and carrier, for a total count of 2 × 10^9^ CFU/g. During the experiment period, quails in all experimental groups had free access to water and feed.

### 2.2. Feed Analysis

The feed samples were analyzed in duplicate and results are reported in Table 1. Both dry matter (DM) [40] (method 930.15) and ash [40] (method 942.05) were determined according to standard procedures. Fat was determined using the Soxhlet extraction procedure [40] (Method 991.36), crude protein was determined by Kjeldahl N × 6.25 procedure [40] (Method 968.06) as described by De Bellis, et al. [41].

### 2.3. Sample and Data Collection

Quail eggs were collected daily, while feed intake was recorded weekly, calculating the difference between administered and residual feed. The average weight of eggs was multiplied by the percentage of daily egg production. Feed conversion ratio was calculated by dividing egg mass production by feed consumption. In each replication group, 4 eggs were collected and measured: egg shape index, egg albumin percentage, egg yolk index, eggshell percentage, yolk color, and Haugh unit. Additionally, Haugh units were calculated using the following formula: Haugh unit = 100 × log(H + 7.57 − 1.7 × W^0.37^), where H = albumen height (mm) and W = egg weight (g) [42]. Egg yolk color was determined using a Roche Color Fan according to the CIE standard colorimetric system (F. Hoffman-La Roche Ltd., Basel, Switzerland).

There were 4 quails for each replicate group, for a total of 16 quails for each experimental group, which were slaughtered and the carcass yield, intestine, liver, spleen, gizzard, breast, and thighs incidence on live weight were determined for each quail. The carcasses were obtained after the feathers, feet, and visceral organs were removed. The carcasses were kept at 4 °C for 18 h, and cold carcass yields were calculated.

Approximately 5 mL of blood samples were collected from each brachial vein of four randomly selected birds from each replicate group after the birds had been fasted for approximately six hours prior to slaughtering procedures (for each experimental group there were 16 blood samples). In addition to blood, aliquots of serum were extracted using additive-free vacutainers centrifuged at 3000× *g* for 10 min. During sampling, serum samples were kept on ice and protected from light. After sampling, they were stored at −80 °C until analysis. The total lipids, glucose, cholesterol, triglycerides, LDL, and HDL were assessed using standard commercial kits (Pars Azmoon Company; Tehran, Iran) using an Anision-300 auto-analyzer system (Shenzhen, Guangdong, China). Malondialdehyde and superoxide dismutase levels were assessed as indicators of antioxidant status.

### 2.4. Statistical Analysis

The data set was analyzed in a completely randomized statistical design according to the General Linear Model (GLM) procedure. Two-way ANOVA was performed to differentiate all the parameters investigated between each treatment. The ANOVA was performed according to the following model:

Y*_ijk_* = µ + A*_i_* + B*_j_* + AB*_ij_* + ε*_ijk_*

*i*: colostrum consumption level.
*j*: probiotics consumption level.
*k*: number of repetitions (*k*= 1, …,4).

where Y*_ijk_* is the observations related to each of the traits studied, µ is the overall mean, A*_i_* is the effect of the *i*th colostrum level (*I* = 1, …,3), B*_j_* is the effect of the *j*th probiotic level (*j* = 1, 2), AB_ij_ is the interaction of the *i*th colostrum level with the *j*th probiotic level, and ε*_ijk_* is the random error effect with zero mean and variance $\;\delta^{2}$.

Significance was set at *p* < 0.05, and the results were expressed as means and mean standard error. All the analyses were performed using SAS 9.2 (Cary, NC, USA) software [43].

## 3. Results

The effects of using different levels of bovine colostrum and probiotics and their interactions on the egg production performance of laying quails are given in Table 2.

The probiotic was not able to affect all investigated traits (*p* > 0.05), while colostrum and its interaction with the probiotic represented factors affecting all of them (*p* < 0.0001). Egg weight and egg mass showed higher values in Col-4 groups, both with and without probiotic addition (*p* < 0.01), while the lowest values of both these parameters were recorded in Col-0 groups (*p* < 0.01). Egg production and feed evaluation ratio has increased when colostrum was included in the diet, independently of its concentration (*p* < 0.01). In the Col-2 and Col-4 groups, without probiotic administration, feed intake was higher than in the Col-0 groups (*p* < 0.05).

Table 3 shows the effects of different levels of bovine colostrum and probiotics, and their interactions, on egg traits. The probiotic was not able to affect all the investigated traits (*p* > 0.05), while colostrum and its interaction with the probiotic was able to affect all of them (*p* < 0.01). Egg albumin percentage, egg yolk index, eggshell percentage, and shell thickness increased with the addition of colostrum (*p* < 0.01), with highest values in Col-4 group with the addition of probiotics (*p* < 0.01); the egg yolk percentage decreased when colostrum was supplemented in the diet (*p* < 0.01). Yolk color and Haugh unit values were higher in Col-4 groups (*p* < 0.01).

The effects of using different levels of bovine colostrum and probiotics and the interactions between them on quail carcass traits are given in Table 4. No effect of the probiotic was observed (*p* > 0.05), while colostrum and its interaction with the probiotic affected all investigated parameters (*p* < 0.01). Carcass percentage was higher in Col-4 quails (*p* < 0.01), while the lowest were observed in Col-0 animals (*p* < 0.01). The highest intestine, gizzard, breast, and thigh percentages were observed in Col-4 groups, both independently of probiotic administration (*p* < 0.01). On the other hand, liver and spleen weight incidence was higher in Col-0 animals (*p* < 0.01).

The effects of using different levels of bovine colostrum and probiotics and the interaction between them on quail serum parameters are shown in Table 5. The probiotic alone did not affect the investigated traits (*p* > 0.05), but on the other hand, colostrum and its interaction with the probiotic did (*p* < 0.01), excluding uric acid, AST, and ALT (*p* > 0.05). The SOD activity and albumin concentration were highest in Col-4 groups, and lowest in Col-0 groups (*p* < 0.01). The total proteins, LDL and Mg concentrations increased when colostrum was added, independently of probiotic inclusion (*p* < 0.01). The Col-4 groups had the lowest MDA and *p* values (*p* < 0.01). The TG increased in Col-4 groups compared to Col-0 (*p* < 0.01); calcium values were higher in Col-2 animals without probiotics compared to all other experimental groups (*p* < 0.01).

## 4. Discussion

A diet serves not only as a source of nutrients for metabolic functions, but also modulates several physiological processes. For newborns, colostrum is crucial to their development because it contains amino acids, proteins, immunoglobulins, growth factors, fatty acids, minerals, and vitamins [44,45]. Moreover, antioxidant, antimicrobial, and immunomodulatory substances represent useful bioactive compounds in colostrum [46,47]. Probiotics are beneficial microorganisms which can be suitably harnessed by food manufacturers and improve animal health and production [33]. Although quails cannot benefit from the immune activity of immunoglobulins from colostrum, the use of it as a feed additive in diets seems useful because of its nutritional and performance-enhancing properties. In this study, the addition of colostrum significantly affected some egg production traits such as average egg weight, percentage of eggs, and mass productions, but also increased feed intake and feed conversion, as well as efficiency in laying quail.

The observed improvement in the performance of laying quails is consistent with previous reports. In a report, it was stated that the use of 50 g/kg of cow’s colostrum powder in the diet of broiler chickens, though it did not have a significant effect on the amount of feed consumed and weight gain of chickens, improved their feed conversion ratio [26]. There is no positive effect of colostrum on the amount of feed consumed and weight gain of chickens in this experiment, due to the low consumption of colostrum in the diet.

The positive effects of using cow colostrum in the diet on broiler chicken performance are consistent with the findings of other research [25,27]. In another report, it was determined that the use of colostrum powder up to 5% in the diet of laying quails improved feed consumption, egg production, egg weight, and feed conversion ratio [48]. These reports are consistent with the findings of the present experiment. Moreover, it was reported that probiotics can improve general health status, FCR, and growth in broilers [49,50,51] and quails [34], although some other authors reported that probiotics were not able to improve hens’ [52] and quails’ [53,54] performances.

The positive effects of bovine colostrum on the diet of laying quails could be due to different reasons. First of all, it has been speculated that an improved in feed intake with fresh colostrum may be related to physical conditioning and the improvement of feed palatability [55]. Another reason for increased performance may be related to the nutritive (proteins, carbohydrates, fats, vitamins, and minerals) and bioactive compounds (antimicrobial factors, antioxidant factors, and immunoglobulins) in colostrum. In some studies carried out on humans, it has been reported that cow colostrum increased muscle growth, accelerated muscle–skeleton regeneration, and enhanced power and strength [56]. Considering the number of research studies that reported similar results in birds, we can speculate that this could be explained by essential and non-essential amino acids, fatty acids, minerals and vitamins, and cellular and tissue growth factors [44,57]. Moreover, the addition of a probiotics supplement could have a synergistic effect with colostrum. Although the normal intestinal microflora is characterized by a stable system [58,59], when intensive farming and environmental stressors occurs, they can lead to nutrient deficiencies that can impair their physiological equilibrium [60,61]. Among other things, probiotics play a role in: the formation of local and systemic immune resistance; forming associations with intestinal mucosa; having interactions with immune system cells and M-cells of Peyer’s patches; the activation of humoral mechanisms to stimulate the immune system and stimulate cell-mediated immunity [62]; the proliferation and differentiation of immune cells which can be stimulated by endothelial cells in the intestinal tract, cytokine production, and other factors [36]; increasing immunoglobulin synthesis both locally and systemically [35]; and in digestion efficiency by stimulating intestinal contractility. Moreover, they have the ability to enhance the cytotoxic functions of T lymphocytes, macrophages, and killer cells [63], as well as play a role in the process of developing food tolerances. The use of diets enriched with probiotics, particularly *Lactobacilli*, is one of the best ways to maintain normal microbial composition and biochemical activity, which promotes immunity, prevents diseases, and improves nutrient absorption. [34].

In this research, probiotics did not improve quail performance. The lack of a positive effect of probiotic consumption on quail performance can be due to reasons such as insufficient consumption, the quality of probiotics consumed, how it is mixed with the diet and the duration of probiotic consumption in quails’ diet. This result is contrary to other findings [51], which show that probiotics in the diet have positive effects on broiler performance, while in other reports, probiotics cannot change laying hen performance [52,53].

Colostrum contains approximately 1 g/L of dietary oligosaccharides per liter, which is twice the amount present in mature milk [64], and can act as a prebiotic because so many are found in the upper intestinal tract. In the intestine, they are not digested and are transported intact to the colon, where they are metabolized by colonic bacteria [65].

Additionally, colostrum is rich in glycosylated proteins (a protein that is glycosylated by adding glucose molecules to amino acid chains) which have been linked to the function of acting as prebiotics by removing saccharin from the gut microbiome by bacteria glycosidases. Colostrum contains a large amount of bovine glycomacropeptide (GMP), which is formed during digestion from casein proteolysis. Bovine GMP has bifidogenic abilities, as demonstrated by concentration-dependent growth enhancement of *Bifidobacterium longum* subspecies [66].

Based on the results obtained from this research, which showed that the use of probiotics along with colostrum did not have significant effects on performance, egg quality traits, carcass traits, antioxidant status, and blood parameters in laying Japanese quails, probably due to the presence of oligosaccharides and glycolyzed proteins in colostrum, which, acting as prebiotics, have improved the function of the intestinal microbiome and affected the effect of probiotics added to quail feed.

The nutritive and non-nutritive elements in cow’s colostrum helped to improve the health status, intestinal morphology, and digestion and absorption of the quails. These elements ultimately contributed to the improvement of their performance.

Based on the results of this research, in laying quails, the use of cow colostrum, in comparison with diets without colostrum, had significant effects on egg quality traits, which is in agreement with the findings of the previous report that powdered colostrum at the level of 2.5 and 5% has been used in the feed of laying Japanese quails. It is the same as increasing feed consumption, egg production, egg weight, eggshell percentage, shell thickness, and improving feed conversion ratio [4]. In the earlier study, there was no change in the volume unit and yolk color. However, in the present report, there is a significant difference in the volume unit and yolk color in quails that used colostrum in their feed (*p* < 0.01). The increase in the percentage of egg albumin, yolk index, percentage and thickness of eggshell, yolk color, and Haugh unit could be related to the richness of colostrum in nutrients, which has improved these traits by absorbing more [48]. The decrease in yolk percentage was due to the increase in white and shell percentages. The increased egg albumin is due to the high percentage of colostrum protein, improvement in eggshell traits is related to the richness of bovine colostrum in terms of calcium, egg yolk is more colorful due to the high content of vitamin A and other useful pigments in colostrum as xanthophyll or carotene [67]. The increase in qualitative traits in this research is consistent with the findings of the report [27,55] on colostrum use in broiler chickens.

Quails have more carcass, intestine, gizzard, breast, and thigh components because cow’s colostrum is rich in nutrients, especially proteins and amino acids. The liver and spleen produce immune cells in the body. The fact that their size does not increase with the age of the quails during this experiment can be due to the fact that the quails receive immunogenic substances through cow colostrum which reduces the pressure on these organs and the body’s lack of endogenous immune cells. Therefore, these two members were less active and small. Regarding cow colostrum’s effects on carcass traits, it is consistent with other reports [27]. An experiment of colostrum supplementation (5% by weight) in the feed of broilers, two weeks after hatching, showed an increase in the feed conversion ratio and improvement of carcass traits in the colostrum supplemented group [26]. A similar effect was observed in young broilers under heat stress given colostrum supplements, as compared with the control group [27].

Reactive oxygen species are a correct characteristic of normal cell activity, such as the mitochondrial respiratory chain, phagocytosis, arachidonic acid metabolism, ovulation, and fertilization. Their production increases several times under pathological conditions [68]. Any situation that leads to an increase in oxidative free radicals (ROS) or a decrease in antioxidant action or incomplete removal of ROS is known as oxidative stress. ROS are toxic to cells (disease caused by bacterial toxins) by inactivating enzymes, denaturing proteins, damaging DNA, and peroxiding lipids [69]. These events lead to cell membrane damage and an increase in active aldehydic substances, such as malondialdehyde, and MDA, which disrupt cell membrane structure and function [70].

Among its enzymatic antioxidants, bovine colostrum contains lactoperoxidase, superoxide dismutase, catalase, as well as non-enzymatic antioxidants, vitamin E, C, and lactoferrin [15,16]. In addition to increasing superoxide dismutase levels, the use of superoxide dismutase in quail diets reduced the production of malondialdehyde in the liver (Table 5).

Superoxide dismutase is an endogenous enzyme and represents the first defense against free radicals. It protects the organism from the harmful effects of oxidants by transforming superoxide radicals, which cause cell injury, into less harmful hydrogen peroxide and molecular oxygen [71]. Studies conducted on mice fed with colostrum reported higher SOD activity [72]. As well as a greater antioxidant activity, it also resulted in a reduced oxidative one, with a strong reduction in MDA production. Vitamin E, one of the most important colostrum compounds with antioxidant activity, which accumulates primarily in cell membranes, strongly inhibits MDA formation [73]. An indicator of lipid peroxidation caused by reactive oxygen species, it is a final product of lipid peroxidation [16].

Different results are reported in the literature regarding uric acid and albumin concentration, with different species involved, different colostrum sources, and different doses. Because the fat content of bovine colostrum is very high, its use in quail diets has increased the LDL and TG serum concentrations, as observed in broilers by Arjomand, Nobakht, and Mehmannavaz [55]. However, other authors reported the opposite results, with a reduction in LDL serum levels in quails consuming colostrum [27]. These contrasting results may be due to the different colostrum sources as well as the age of the investigated animals. As colostrum contains high levels of saturated fats, and newborn chicks have serious limitations in fat digestion (an especially saturated form of them), fat could not be effectively digested which could alleviate some blood-related agents such as total cholesterol and LDL. Freeze and souring may be related to fat sources treated in colostrum and may affect their digestion capacity [55].

## 5. Conclusions

The use of colostrum and probiotics in the feeding of adult quails has a positive effect on their egg production, egg quality, growth, carcass characteristics’ productivity, health status, and preservation. In particular, introducing colostrum into the diet at 4% can improve egg quality traits, antioxidant status, as well as blood indicators, whereas probiotics had no effects in these fields. In order to better understand these results and improve knowledge on other aspects, such as meat production, deeper studies are required.

## Figures and Tables

**Table 1:** Composition of experimental diets.

Feed Ingredients (%)	Colostrum Inclusion					
	0-Col/Pro-0	Col-2/pro-0	Col-4/pro-0	Col-0/pro-0.01	Col-2/pro-0.01	Col4/pro-0.01
Corn	50.00	49.00	48.00	50.00	49.00	48.00
Wheat	23.73	22.18	20.63	23.73	22.18	20.63
Soybean meal-44%	16.31	14.75	13.21	16.31	14.75	13.21
Soybean oil	0.24	0.36	0.48	0.24	0.36	0.48
Colostrum	0	2.00	4.00	0	2.00	4.00
Bio-Poul^®^	0	0	0	0.01	0.01	0.01
Oyster shell	7.83	7.83	7.81	7.82	7.83	7.81
Dicalcium phosphate	1.11	1.10	1.10	1.11	1.10	1.10
Salt	0.28	0.28	0.27	0.28	0.28	0.27
Vitamin premix ^1^	0.25	0.25	0.25	0.25	0.25	0.25
Mineral premix ^2^	0.25	0.25	0.25	0.25	0.25	0.25
Calculated composition						
Metabolisable energy (Kcal/kg)	2800	2800	2800	2800	2800	2800
Crude protein (%)	14.00	14.00	14.00	14.00	14.00	14.00
Ether extract (%)	3.70	3.64	3.59	3.70	3.64	3.59
Ca (%)	3.28	3.28	3.28	3.28	3.28	3.28
Available phosphor (%)	0.31	0.31	0.31	0.31	0.31	0.31
Crude fiber (%)	2.86	3.28	3.98	2.86	3.28	3.98
Sodium (%)	0.15	0.15	0.15	0.15	0.15	0.15
Lysine (%)	0.63	0.63	0.63	0.63	0.63	0.63
Methionine + Cysteine (%)	0.55	0.55	0.55	0.55	0.55	0.55
Tryptophan (%)	0.18	0.18	0.18	0.18	0.18	0.18

^1^ Vitamin premix per kg of diet: vitamin A (retinol), 8,500,000 IU; vitamin D3 (Cholecalciferol), 2,500,000 IU; vitamin E (tocopheryl acetate), 11,000 IU; vitamin k_3_, 2200 mg; thiamine, 1477 mg; riboflavin, 4000 mg; panthothenic acid, 7840 mg; pyridoxine, 7840 mg; cyanocobalamin, 10 mg; folic acid, 110 mg; choline chloride, 400,000 mg. ^2^ Mineral premix per kg of diet: Fe (FeSO_4_·7H_2_O, 20.09% Fe), 75,000 mg; Mn (MnSO_4_·H_2_O, 32.49% Mn), 74.4 mg; Zn (ZnO, 80.35% Zn), 64.675 mg; Cu (CuSO_4_·5H_2_O), 6000 mg; I (KI, 58% I), 867 mg; Se (NaSeO_3_, 45.56% Se), 200 mg.

**Table 2:** The effect different levels of bovine colostrum and probiotics on performance of laying quails.

	Prob-0 ^1^			Prob-0.01 ^2^			MSE ^3^	Analysis of Variance		
	Col-0	Col-2	Col-4	Col-0	Col-2	Col-4		P ^4^	C ^5^	P × C
Egg weight (g)	11.57 ^c^	12.09 ^b^	12.44 ^a^	11.66 ^b^	11.88 ^b^	12.54 ^a^	0.10	0.9136	>0.05	>0.05
Egg production (%)	51.13 ^b^	67.22 ^a^	69.88 ^a^	52.79 ^b^	67.26 ^a^	72.54 ^a^	1.69	0.5581	>0.05	>0.05
Egg mass (g/h/d)	5.91 ^c^	8.15 ^b^	8.76 ^a^	6.15 ^b^	8.00 ^b^	9.03 ^a^	0.21	0.6704	>0.05	>0.05
Feed intake (g/h/d)	24.71 ^b^	26.06 ^a^	26.14 ^a^	24.55 ^b^	25.92 ^ab^	26.03 ^a^	0.29	0.4495	>0.05	>0.05
FCR ^6^	4.23 ^a^	3.40 ^b^	3.18 ^b^	4.07 ^a^	3.44 ^b^	3.02 ^b^	0.09	0.3771	>0.05	>0.05

^1^ Prob-0 = 0% of probiotics inclusion; ^2^ Prob-0.01 = 0.01% of probiotics inclusion; ^3^ MSE = Mean standard error; ^4^ P = Probiotic; ^5^ C = Colostrum; ^6^ FCR = Feed Conversion Ratio; Values in the same row not sharing a common superscript differ significantly (*p* < 0.01).

**Table 3:** The effect of different levels of bovine colostrum and probiotics on egg traits of laying quails.

	Prob-0 ^1^			Prob-0.01 ^2^			MSE ^3^	Analysis of Variance		
	Col-0	Col-2	Col-4	Col-0	Col-2	Col-4		P ^4^	C ^5^	P × C
Egg albumin (%)	59.98 **^c^**	62.86 **^ab^**	63.41 **^ab^**	62.15 **^b^**	62.40 **^b^**	64.77 **^a^**	0.38	0.9000	0.0001	0.0001
Egg yolk index	60.07 **^c^**	61.72 **^bc^**	65.63 **^ab^**	59.00 **^c^**	60.76 **^bc^**	67.75 ^a^	0.52	0.1340	0.0001	0.0003
Egg yolk (%)	30.76 **^a^**	27.75 **^b^**	28.81 **^b^**	29.03 **^ab^**	28.46 **^b^**	27.30 **^b^**	0.43	0.9857	0.0041	0.0006
Eggshell (%)	8.57 **^c^**	9.28 **^ab^**	9.40 **^ab^**	8.80 **^bc^**	9.25 **^ab^**	9.71 **^a^**	0.14	0.3940	0.0001	0.0003
Shell Thickness (mm)	0.238 **^c^**	0.255 **^bc^**	0.285 **^bc^**	0.245 **^c^**	0.258 **^bc^**	0.300 ^a^	0.01	0.4615	0.0001	0.0003
Yolk color	3.5 **^c^**	4.79 **^b^**	7.21 **^a^**	3.5 **^c^**	5.30 **^b^**	7.16 **^a^**	0.28	0.8223	0.0001	0.0001
Haugh unit	95.52 **^b^**	96.88 **^b^**	99.68 **^a^**	95.18 **^b^**	96.28 **^b^**	99.77 **^a^**	0.51	0.7507	0.0001	0.0001

^1^ Prob-0 = 0% of probiotics inclusion; ^2^ Prob-0.01 = 0.01% of probiotics inclusion; ^3^ MSE = Mean standard error; ^4^ P = Probiotic; ^5^ C = Colostrum; Values in the same row not sharing a common superscript differ significantly (*p* < 0.01).

**Table 4:** The effect of different levels of bovine colostrum and probiotics on carcass traits of laying quails (%).

	Prob-0 ^1^			Prob-0.01 ^2^			MSE ^3^	Analysis of Variance		
	Col-0	Col-2	Col-4	Col-0	Col-2	Col-4		P ^4^	C ^5^	P × C
Carcass	64.16 ^bc^	70.05 ^abc^	73.40 ^a^	62.79 ^c^	72.23 ^ab^	73.17 ^a^	2.05	0.2748	0.0001	0.0001
Intestine	7.69 ^b^	7.69 ^b^	11.27 ^b^	7.73 ^b^	8.37 ^b^	11.43 ^a^	0.42	0.8051	0.0001	0.0001
Liver	6.59 ^a^	4.05 ^b^	3.60 ^b^	5.85 ^a^	4.12 ^b^	3.65 ^b^	0.2	0.6834	0.0001	0.0001
Spleen	0.30 ^a^	0.16 ^b^	0.16 ^b^	0.29 ^a^	0.20 ^b^	0.15 ^b^	0.02	0.7586	0.0001	0.0001
Gizzard	3.65 ^b^	4.12 ^ab^	5.03 ^a^	3.45 ^b^	4.13	4.82 ^a^	0.24	0.6566	0.0001	0.0009
Breast	30.83 ^b^	33.05 ^b^	36.17 ^a^	30.72 ^b^	34.70 ^b^	35.57 ^ab^	1.14	0.8036	0.0005	0.0096
Thighs	16.79 ^bc^	19.19 ^ab^	20.32 ^a^	15.31 ^c^	19.32 ^ab^	20.18 ^a^	0.57	0.5831	0.0001	0.0001

^1^ Prob-0 = 0% of probiotics inclusion; ^2^ Prob-0.01 = 0.01% of probiotics inclusion; ^3^ MSE = Mean standard error; ^4^ P = Probiotic; ^5^ C = Colostrum; Values in the same row not sharing a common superscript differ significantly (*p* < 0.01).

**Table 5:** The effect of different levels of bovine colostrum and probiotics on blood indexes of laying quails.

	Prob-0 ^1^			Prob-0.01 ^2^			MSE ^3^	Analysis of Variance		
	Col-0	Col-2	Col-4	Col-0	Col-2	Col-4		P ^4^	C ^5^	P × C
SOD ^6^ (g/dL)	1.35 ^c^	2.68 ^b^	3.44 ^a^	1.33 ^c^	2.68 ^b^	3.41 ^a^	0.15	0.9606	0.0001	0.0001
Uric acid (g/dL)	4.38	4.35	4.74	4.28	4.05	4.96	0.24	0.9013	0.0141	0.1178
Albumin (g/dL)	1.25 ^c^	1.53 ^abc^	1.62 ^ab^	1.33 ^bc^	1.57 ^abc^	1.65 ^a^	0.07	0.6813	0.0001	0.0025
Protein (g/dL)	3.43 ^b^	4.10 ^a^	4.25 ^a^	3.43 ^b^	4.15 ^a^	4.35 ^a^	0.16	0.9619	0.0001	0.0009
ALT ^7^ (mg/dL)	32.50 ^b^	31.25 ^b^	33.25 ^a^	32.00 ^b^	34.00 ^a^	33.25 ^a^	2.31	0.4644	0.8039	0.9926
AST ^8^ (mg/dL)	245.50	253.25	260.00	245.00	275.50	237.50	12.17	0.9868	0.7103	0.7820
MDA ^9^ (g/dL)	4.20 ^a^	3.83 ^b^	1.93 ^c^	4.20 ^a^	2.00 ^c^	1.53 ^d^	0.38	0.1492	0.0001	0.0001
LDL ^10^ (mg/dL)	38.35 ^b^	48.95 ^a^	48.45 ^a^	38.45 ^b^	47.90 ^a^	51.45 ^a^	1.69	0.8935	0.0001	0.0001
TG ^11^ (mg/dL)	765.00 ^b^	979.00 ^a^	989.00 ^a^	769.00 ^b^	958.00 ^a^	1029.00 ^a^	35.34	0.8135	0.0001	0.0001
Ca (mg/dL)	29.50 ^b^	35.38 ^a^	27.60 ^a^	29.00 ^b^	25.00 ^c^	23.20 ^c^	1.26	0.9684	0.0001	0.0038
P (mg/dL)	7.28 ^a^	5.84 ^ab^	5.70 ^b^	6.68 ^ab^	5.64 ^b^	5.36 ^b^	0.32	0.3056	0.0003	0.0039
Mg (mg/dL)	3.43 ^a^	2.68 ^b^	2.60 ^b^	3.45 ^a^	2.60 ^b^	2.60 ^b^	0.12	0.9441	0.0001	0.0001

^1^ Prob-0 = 0% of probiotics inclusion; ^2^ Prob-0.01 = 0.01% of probiotics inclusion; ^3^ MSE = Mean standard error; ^4^ P = Probiotic; ^5^ C = Colostrum; ^6^ SOD = superoxide dismutase; ^7^ ALT = Alanine aminotransferase; ^8^ AST = aspartate aminotransferase; ^9^ MDA = Malondialdehyde; ^10^ LDL = Low density lipoprotein; ^11^ TG = triglycerides; Values in the same column not sharing a common superscript differ significantly (*p* < 0.01).

## Data Availability

Data sharing not applicable.
